# Novelties in Vietnamese *Staurogyne* (Acanthaceae): *S. konchurangensis*, a new species, and amended description of *S. purpurea*

**DOI:** 10.3897/phytokeys.279.210807

**Published:** 2026-09-10

**Authors:** Le Ngoc Han, Bui Hong Quang, Khang Sinh Nguyen, Maxim S. Nuraliev, Ritesh Kumar Choudhary, Duong Thi Hoan, Do Van Hai

**Affiliations:** 1 Institute of Biology, Vietnam Academy of Science and Technology, 18, Hoang Quoc Viet road, Nghia Do, Hanoi 10072, Vietnam Institute of Biology, Vietnam Academy of Science and Technology Hanoi Vietnam; 2 Graduate University Science and Technology, Vietnam Academy of Science and Technology, 18, Hoang Quoc Viet road, Nghia Do, Hanoi 10072, Vietnam Graduate University Science and Technology, Vietnam Academy of Science and Technology Hanoi Vietnam; 3 Joint Vietnam-Russia Tropical Science and Technology Research Center, A.N. Severtsov Institute of Ecology and Evolution, Russian Academy of Sciences, 33, Leninsky avenue, Moscow 119071, Russia Joint Vietnam-Russia Tropical Science and Technology Research Center, A.N. Severtsov Institute of Ecology and Evolution, Russian Academy of Sciences Moscow Russia; 4 Department of Higher Plants, Faculty of Biology, M.V. Lomonosov Moscow State University, 1, 12, Leninskie Gory, Moscow 119234, Russia Department of Higher Plants, Faculty of Biology, M.V. Lomonosov Moscow State University Moscow Russia; 5 Department of Botany, University of Delhi, Delhi 110007, India Department of Botany, University of Delhi Delhi India

**Keywords:** Conservation, Gia Lai Province, Ha Giang Province, morphology, plant diversity, taxonomy, Tuyen Quang Province

## Abstract

*Staurogyne konchurangensis* (Acanthaceae) is described and illustrated from Vietnam (Gia Lai Province, Kon Chu Rang Nature Reserve) as a species new to science. It is distinguished from its congeners by the following combination of morphological characters: bracts 5–8 mm long, calyx 10–13 mm long, and white corolla ca. 1.5–2 cm long. In addition, an amended description of *S. purpurea* is presented based on newly collected specimens and field observations; its key diagnostic features and geographical distribution are clarified.

## Introduction

*Staurogyne* Wall. is a species-rich genus belonging to the subfamily Nelsonioideae of Acanthaceae (Scotland and Vollesen 2000). Nelsonioideae consists of 6 genera and ca. 175 species and is characterized by the absence of cystoliths in epidermal cells, descending cochlear aestivation of corolla lobes, more than four ovules per ovary, and albuminous seeds which lack funiculi (Scotland and Vollesen 2000; McDade et al. 2012; Daniel and McDade 2014). *Staurogyne*, the largest genus of Nelsonioideae, comprises around 166 species distributed in tropical and subtropical regions of America, Africa and Asia (Daniel and McDade 2014; POWO 2026). It is characterized by having bracteoles attached to the pedicel (i.e. not adnate to the receptacle or to the base of the calyx tube), subequal or unequal calyx lobes, corollas usually less than 3 cm long, two stamens or four didynamous stamens per flower, and capsules lacking a retinacula and containing numerous minute seeds (Lindau 1895; Benoist 1933, 1935; Bremekamp 1965; Hansen 1985; Scotland et al. 1994; Scotland and Vollesen 2000; Daniel and McDade 2014; Braz and Monteiro 2017; Choopan et al. 2019; Manzitto-Tripp et al. 2022). In Vietnam, 27 species of *Staurogyne* have been recorded (Lien 2005; Hai and Khoi 2015; Hai et al. 2018).

This paper further contributes to the knowledge of *Staurogyne* in Vietnam. Here, we describe *Staurogyne konchurangensis*, a species new to science, and present for the first time a comprehensive account of *S. purpurea* based on investigation of newly collected specimens. For each of these species, information on distribution, ecology and phenology are provided, conservation status is assessed, and morphological comparison with the most similar congeners is given.

## Materials and methods

*Staurogyne konchurangensis* was collected during fieldwork in Kon Chu Rang Nature Reserve (Gia Lai Province) by the team of D.V. Hai in July and September 2017 and by the team of M.S. Nuraliev in May 2016 and November 2022. *Staurogyne purpurea* was collected during fieldwork by the team of B.H. Quang in Tuyen Quang Province: in Tung Vai Commune in April 2024, and in Minh Tan Commune in April 2025 and June 2026 (previously included in Ha Giang Province).

The morphological descriptions are based on the examination of fresh and dried specimens. Measurements were made on freshly collected materials. Voucher specimens were prepared following the standard protocols (Davies et al. 2023) and are deposited in HN, LE, and MW. Terminology follows Beentje (2016). Photographs were taken using Canon EOS 6D Mark II and Pentax Optio W80 digital cameras.

Relevant specimens of *Staurogyne* from Vietnam and neighboring countries were examined in various herbaria through high-resolution digital images available via the Global Plants on JSTOR (https://plants.jstor.org/) and the Global Biodiversity Information Facility (GBIF; https://www.gbif.org). The conservation status was assessed following the IUCN Red List Categories and Criteria (IUCN 2024), based on field observations and herbarium specimen data.

## Taxonomic treatment

### Staurogyne konchurangensis

Taxon classificationPlantaeLamialesAcanthaceae

D.V.Hai & Nuraliev sp. nov.

8CB18BE1-2C66-55F9-8F7C-111B76273C9E

urn:lsid:ipni.org:names:77396493-1

Figs 1, 2, 3

#### Type.

Vietnam • Gia Lai Province: Son Lang Commune, Kon Chu Rang Nature Reserve, 14°30'46.7"N, 108°32'46.6"E, 986 m a.s.l., 19 September 2017, *D.V. Hai, T.D. Binh, L.N. Han, B.H. Quang, D.H. Son KCR 317* (holotype HN!; isotypes HN!).

#### Diagnosis.

Similar to *Staurogyne concinnula* (Hance) Kuntze and *S. debilis* (T.Anderson) C.B.Clarke in habit (herbs about 10–20 cm tall), lax racemes, and white corolla. It is distinguished from *S. concinnula* by its longer bracts (5–8 vs. 2–4 mm), longer calyx (10–13 mm vs. 5–7 mm long), longer corolla (ca. 1.5–2 cm vs. ca. 1 cm long), and larger capsules (8–9 × 2.5–3 mm vs. 4–6 × 1.5 mm). The new species differs from *S. debilis* in having longer (3–9.5 cm vs. 2.5–4 cm) oblanceolate (vs. elliptic, oblong, or oblong-ovate) leaves, larger bracts (5–8 × 1–2 mm vs. 2 × 0.4 mm), longer calyx (10–13 mm vs. 5–6 mm long), longer corolla (ca. 1.5–2 cm vs. ca. 1 cm long), and longer capsules (8–9 × 2.5–3 mm vs. ca. 6 mm long). A detailed comparison of these three species is provided in Table 1.

**Figure 1.**
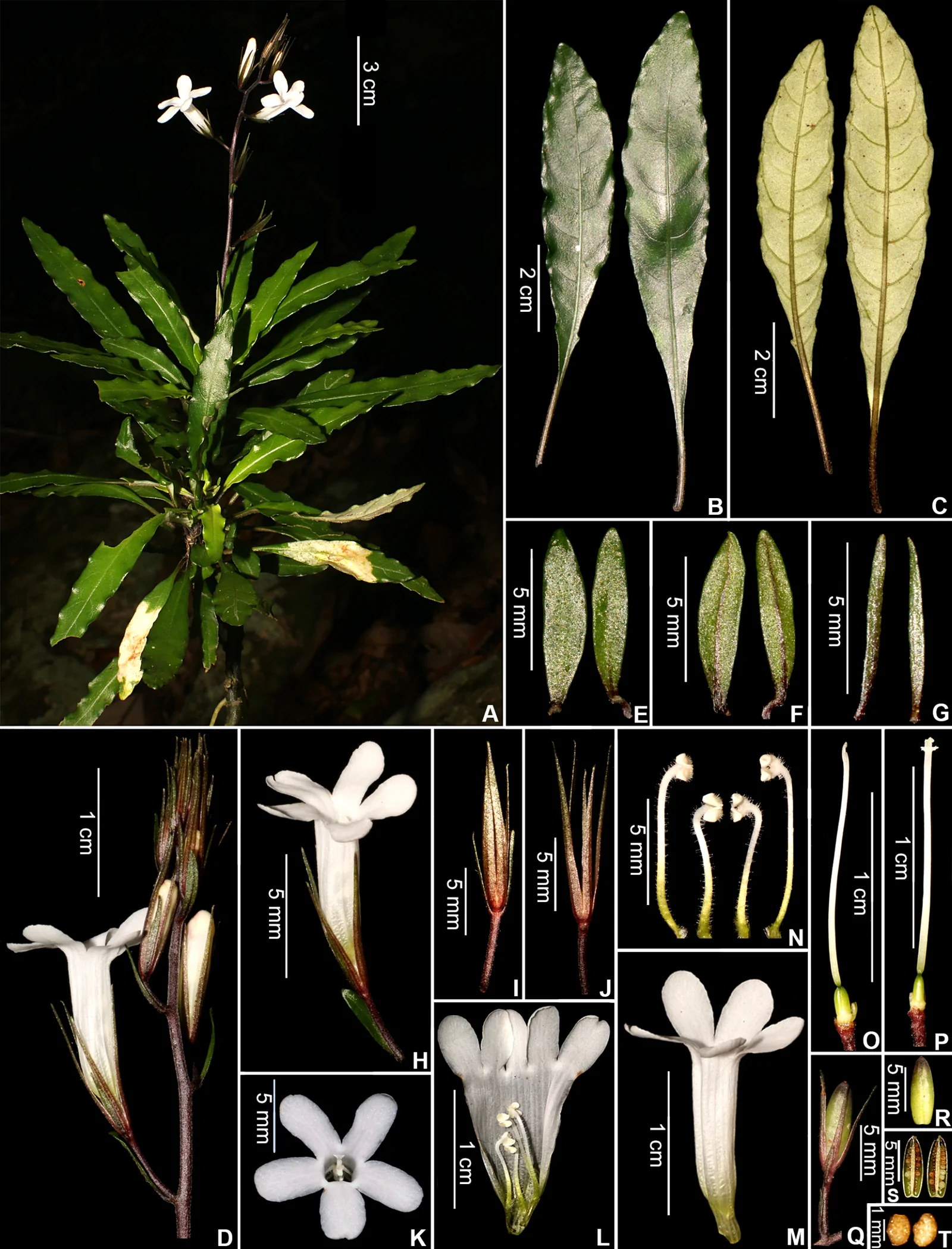
*Staurogyne konchurangensis*. **A**. Flowering branch; **B**. Leaves (adaxial view); **C**. Leaves (abaxial view); **D**. Inflorescence; **E**. Bracts (adaxial view); **F**. Bracts (abaxial view); **G**. Bracteoles; **H**. Flower with bract and bracteoles; **I**. Bracteoles and calyx; **J**. Calyx; **K**. Flower, front view; **L**. Artificially opened corolla; **M**. Corolla (side view); **N**. Stamens (anterior on the sides, posterior in the center); **O, P**. Ovary and style; **Q**. Fruit and calyx; **R**. Fruit; **S**. Artificially opened fruit; **T**. Seeds. Photos by D.V. Hai based on *D.V. Hai et al. KCR 317*.

**Table 1.** Morphological comparison of *Staurogyne konchurangensis*, *S. concinnula*, and *S. debilis*.

**Characters**	***S. konchurangensis***	***S. concinnula***	***S. debilis***
References	this study	Benoist (1935), Yamazaki (1993), Hsieh and Huang (1998), Hu et al. (2011)	Hsieh et al. (1999), Hu et al. (2011)
Stem branching	usually branched	usually unbranched	unbranched or rarely branched
Petiole length	0.5–2.0 cm	0.3–3 cm	0.5–0.8 cm
Leaf blade: shape	oblanceolate	oblanceolate	elliptic, oblong, or oblong-ovate
Leaf blade: size	3–9.5 × 0.7–2.0 cm	1.5–7 × 0.5–2 cm	2.5–4 × 0.8–1.7 cm
Inflorescence: length	6–13 cm	5–15 cm	up to 7 cm
Bracts: size	5–8 × 1–2 mm	2–4 mm long, width unknown	2 × 0.4 mm
Bracteoles: length	5–7 mm	ca. 2 mm	1 mm
Pedicel: length	4–6 mm	1–4 mm	ca. 2 mm
Calyx: length	10–13 mm	5–7 mm	5–6 mm
Calyx: indumentum	almost glabrous but sparsely pubescent on margin and midvein	glabrous except for minutely pilose margins	glabrous except for sparsely hirsute margins
Corolla: length	ca. 1.5–2 cm	ca. 1 cm	ca. 1 cm
Stamens: length	longer pair 7–9 mm and shorter pair 6–7 mm	longer pair ca. 5 mm and shorter pair ca. 2.5 mm	longer pair ca. 3 mm and shorter pair ca. 2 mm
Stamen filaments: indumentum	glandular hairy	glabrous	strigose
Style: length	11–14 mm	ca. 5–8 mm	ca. 8 mm
Stigma: shape	2-lobed	subulate	2-cleft or lobed
Fruit: size	8–9 × 2.5–3.0 mm	4–5 × ca. 1.5 mm	ca. 6 mm long, width unknown

#### Description.

***Herbs*** to 20 cm tall, branching at the stem base or above the base. ***Stems*** stout, terete, pubescent, brown-purple. ***Leaves*** opposite; petiole 0.5–2.0 cm long, pubescent, brown-purple; blade oblanceolate, 3–9.5 × 0.7–2.0 cm, base cuneate and decurrent onto the petiole, margin slightly sinuate and revolute on the abaxial side, apex obtuse; blade adaxially dark green, glabrous, with dark green cystoliths, abaxially whitish green, sparsely pubescent along veins and glabrous otherwise; midvein flat adaxially and raised abaxially; secondary veins 6–8 on each side of the midvein, netted near the margin. ***Racemes*** terminal and axillary, lax, 6–13 cm long, 4–14-flowered; peduncle and rachis pubescent, brown-purple; bracts lanceolate to subulate, 5–8 × 1–2 mm, brown-purple, margin entire, apex acute to obtuse, adaxially glabrous, abaxially sparsely pubescent along veins, midvein flat adaxially and raised abaxially; bracteoles (2 per flower) opposite, attached to the pedicel just below the calyx, linear, 5–7 mm long, both surfaces almost glabrous. ***Pedicel*** 4–6 mm long, pubescent, purple. ***Flower*** weakly zygomorphic. ***Calyx*** 5-lobed nearly to the base, purple; lobes linear, almost glabrous but sparsely pubescent on the margin and midvein, unequal in size; unpaired (adaxial) lobe and lobes of the abaxial pair 10–13 mm long; lobes of the adaxial pair 8–11 mm long. ***Corolla*** ca. 1.5–2 cm long, outside glabrous or shortly pubescent, inside mostly glabrous but with a tuft of hairs at the level of transition between the narrow and wide portions of the tube (i.e. at the level of the stamen attachment), white and sometimes with slight purple tint; tube with a cylindrical proximal portion ca. 3 mm long and 1.0–1.5 mm in diameter, widening to a cylindrical distal portion 11–12 mm long and 3.0–3.5 mm in diameter, mostly straight but slightly bent to the adaxial side at the transitional level; lobes 5, widely spreading (corolla rotate), slightly unequal, oblong, rounded at apex, 4.0–5.0(6.0) × 2.0–3.5 mm; abaxial lobe with an apical incision ca. 0.5 mm deep. ***Stamens*** 4, didynamous, inserted at the transition between the narrow and wide portions of the corolla tube, included, white; posterior (adaxial) stamens 6–7 mm long, anterior (abaxial) stamens 7–9 mm long; filaments almost straight but bent downwards near their apices, glandular hairy; anthers with broad connectives and deeply separated thecae, appressed to the adaxial side of the corolla tube; thecae 1.0 × 0.5 mm, margin glabrous; staminode ca. 0.5 mm long, brown. ***Disk*** annular, white, smooth and lustrous. ***Ovary*** 1–2 mm long, ovoid-oblong, glabrous, green, bilocular; ovules 26–28 per locule, aligned in a row from the base to near to the apex of each locule; style appressed to the adaxial side of the corolla tube, near the base constricted by the bases of the stamen filaments, 11–14 mm long, glabrous, white; stigma unequally 2-lobed, slightly exserted; adaxial stigma lobe 0.5 mm long, apically divided into 2 small lobules, each lobule serrate around apex; abaxial stigma lobe 1.0 mm long, serrate around apex. ***Capsule*** oblong, 8–9 × 2.5–3.0 mm, glabrous, 52–56-seeded; seeds oblong to narrowly ovoid, compressed, 1.0 × 0.7 mm, alveolate.

**Figure 2.**
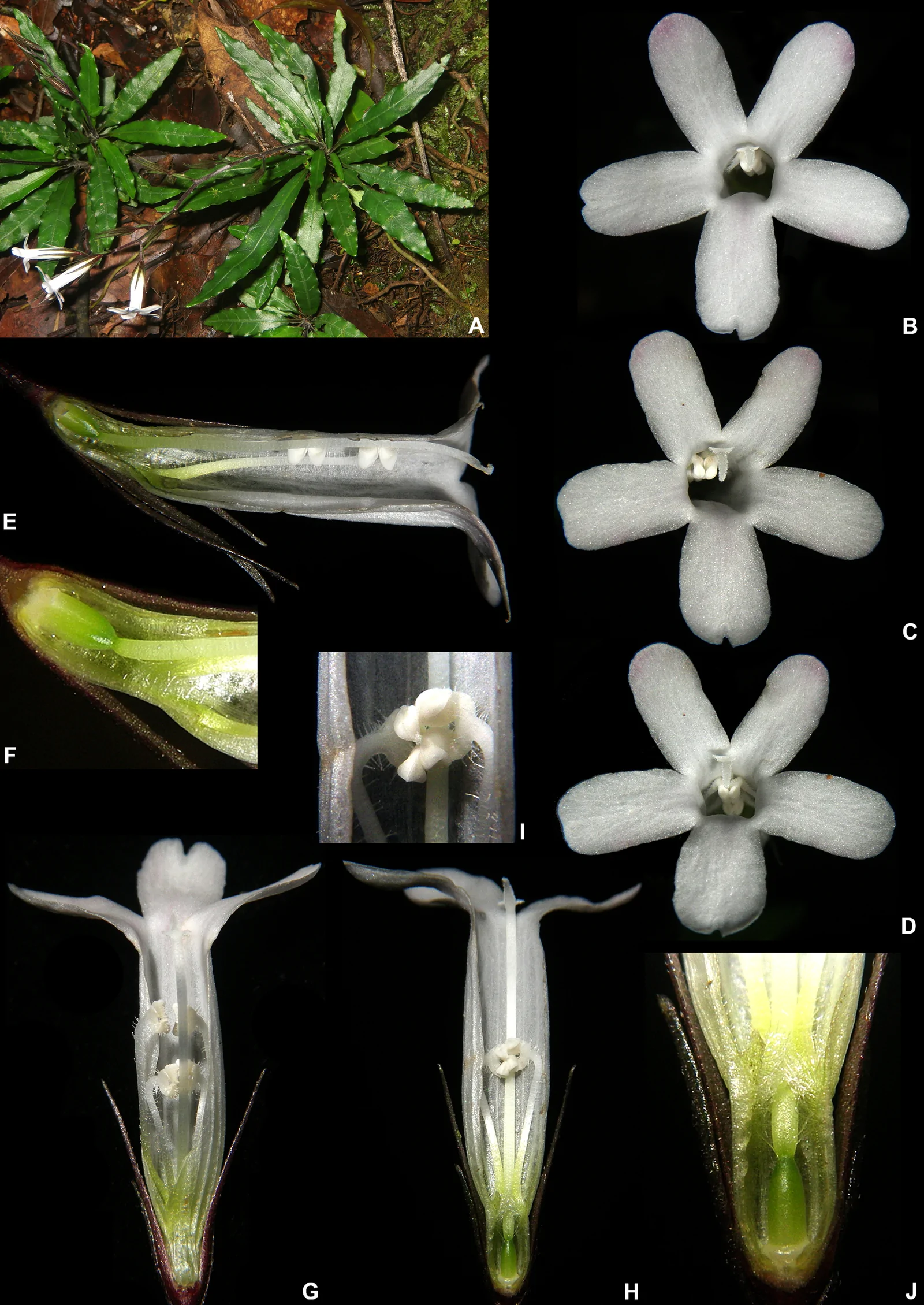
*Staurogyne konchurangensis*, flower morphology. **A**. Habit; **B–D**. Flower (front views); **E**. Longitudinally dissected flower; **F**. Longitudinally dissected proximal portion of flower; **G**. Flower with corolla partly removed on the adaxial side. **H**. Flower with abaxial half of perianth and anterior stamens removed; **I**. Portion of H, showing anthers of the posterior stamens. **J**. Portion of H, showing corolla base, disk, and ovary. Photos by M.S. Nuraliev based on *Nuraliev et al. NUR 3913*.

#### Distribution and habitat.

*Staurogyne konchurangensis* is currently known only from Kon Chu Rang Nature Reserve in Gia Lai Province, Vietnam. It grows under the shade of primary and secondary evergreen broad-leaved forest on fertile soil, along the streams at elevations of 900–1000 m.

**Figure 3.**
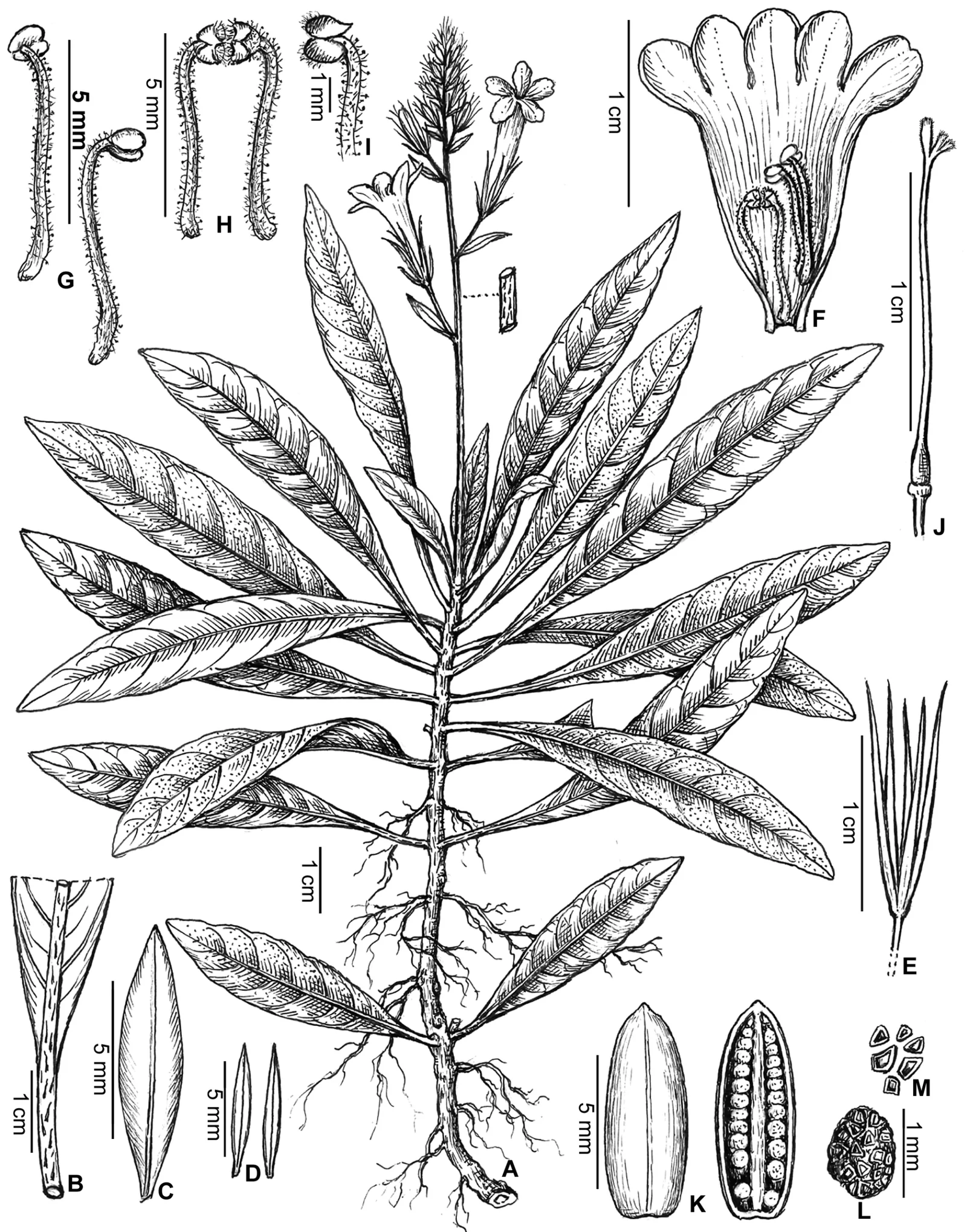
*Staurogyne konchurangensis*. **A**. Flowering plant; **B**. Base of leaf (abaxial view); **C**. Bract; **D**. Bracteoles; **E**. Calyx; **F**. Artificially opened corolla; **G**. Anterior stamens. **H**. Posterior stamens; **I**. Anther (adaxial view); **J**. Ovary and style; **K**. Fruit (outside and inside); **L**. Seed; **M**. Portion of seed surface. Drawn by Le Kim Chi based on *D.V. Hai et al. KCR 317*.

#### Phenology.

Flowering and fruiting from May to November.

#### Etymology.

The species is named after its type locality, Kon Chu Rang Nature Reserve.

#### Vernacular name.

Vietnamese (proposed here): *Nhụy thập kon chư răng*.

#### Conservation status.

*Staurogyne konchurangensis* occurs in a legally protected territory, i.e. the Kon Chu Rang Nature Reserve. It grows at undisturbed sites where only a slight anthropogenic threat has been detected. There are no data on its population size and structure. Therefore, we preliminarily assess its status as ‘Data Deficient’ (DD) according to the IUCN Red List Categories and Criteria (IUCN 2024).

#### Additional specimens examined (paratypes).

Vietnam • Gia Lai Province: Son Lang Commune, Kon Chu Rang Nature Reserve, 29 km ESE of Mang Den town, forest, river bank, 14°31'00"N, 108°32'30"E, 980 m a.s.l., 28 May 2016, *M.S. Nuraliev 1584* (HN; MW MW0595920, MW0595921); • Gia Lai Province: Son Lang Commune, Kon Chu Rang Nature Reserve, 14°30'42.3"N, 108°34'23.2"E, 983 m a.s.l., 26 July 2017, *D.V. Hai, T.D. Binh, D.T. Hoan, K.S. Nguyen, B.H. Quang, D.H. Son KCR 82* (HN); • Gia Lai Province: Son Lang Commune, Kon Chu Rang Nature Reserve, 29 km ESE of Mang Den town, forest, river bank, near waterfall, 14°30'50"N, 108°32'46"E, 1000 m a.s.l., 29 November 2022, *M.S. Nuraliev, C.I. Fomichev, D.F. Lyskov NUR 3913* (MW MW0595922, MW0595923).

#### Taxonomic notes.

*Staurogyne konchurangensis* is readily distinguishable from all its congeners by having brown-purple pubescent stems, lanceolate to subulate brown-purple bracts, and a white corolla approximately 1.5–2 cm long. The differences of the new species from its morphologically closest relatives, *S. concinnula* and *S. debilis*, are outlined in the diagnosis.

#### Notes on morphology.

Flowers of *S. konchurangensis* often appear to have inverted or oblique orientation. While this phenomenon may result from resupination, a condition known in some members of Acanthaceae (e.g. in many Strobilanthinae: Moylan et al. 2004), resupination has never been documented in *Staurogyne*. To find out the reasons for the non-typical flower orientation in this species, further studies are required, including examination of living material. Apart from resupination, it can possibly be a result of the inflorescence rachis and/or pedicel bending.

### Staurogyne purpurea

Taxon classificationPlantaeLamialesAcanthaceae

Aver. & K.S.Nguyen, Pl. Diversity Fl. Veg. Bat Dai Son: 213 (2020)

A083AC8A-5D55-5DCE-9402-8DE0A3CE6B3A

Figs 4, 5, 6

#### Type.

Vietnam • Tuyen Quang Province [previously Ha Giang Province, Quan Ba District]: Tung Vai Commune, Thang village, around point 21°03'13.4"N, 104°51'48.8"E, steep rocky slopes of streams valleys composed of eroded stratified highly eroded limestone, 1000–1200 m, primary evergreen broad-leaved very humid forests, terrestrial herb to 1.2 m tall on steep shady slopes, rare, 23 April 2018, *L. Averyanov, K.S. Nguyen, N.T. Hiep, N.Q. Hieu, C.Q. Ngan, T. Maisak, VR 750* (holotype LE LE01050144!; isotype HN!).

**Figure 4.**
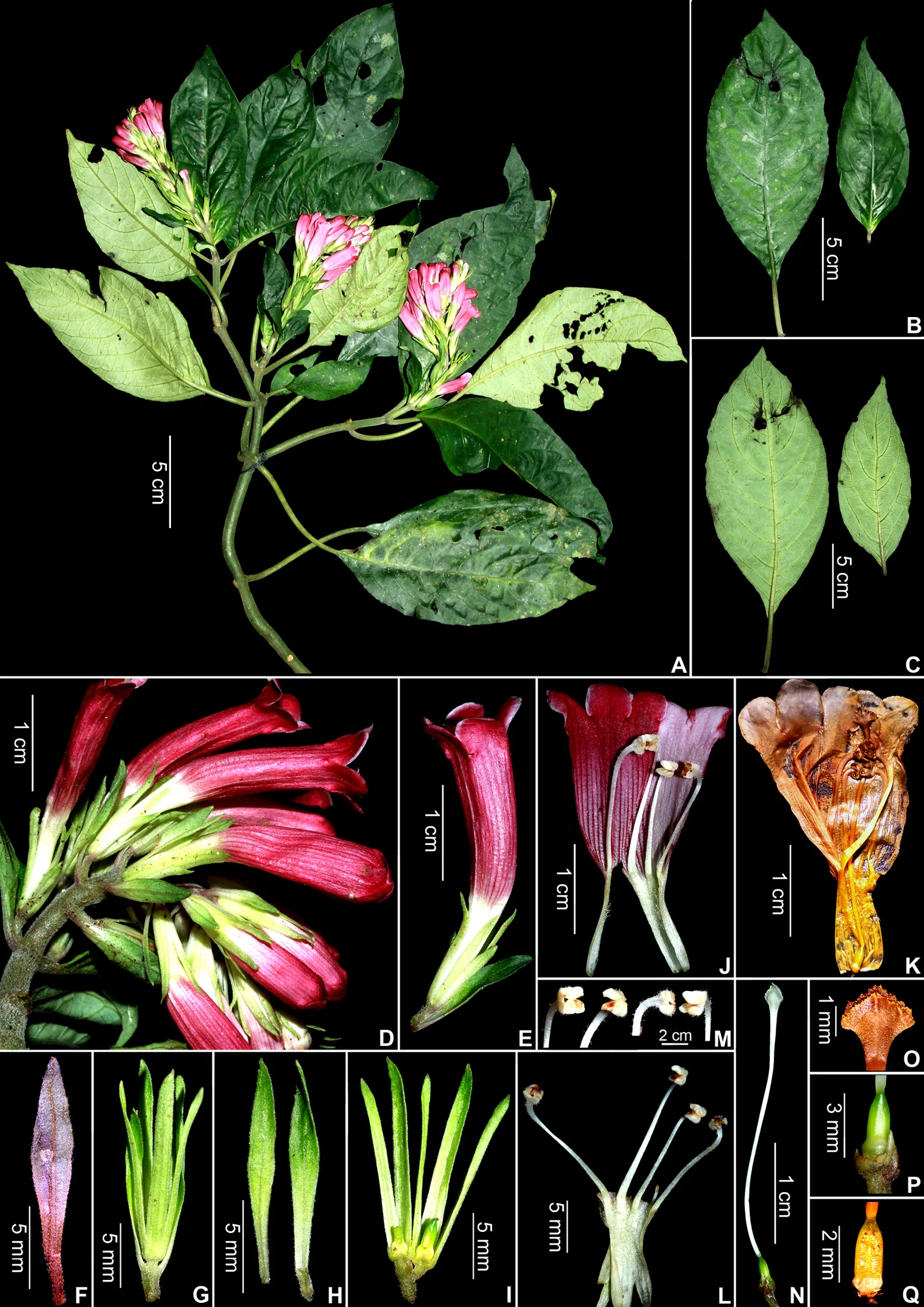
*Staurogyne purpurea*. **A**. Flattened flowering branch; **B**. Leaves (adaxial view); **C**. Leaves (abaxial view); **D**. Inflorescence; **E**. Flower with bract and bracteoles; **F**. Bract (abaxial view); **G**. Bracteoles and calyx; **H**. Bracteoles (adaxial view); **I**. Artificially opened calyx, showing the ovary; **J, K**. Artificially opened corolla; **L**. Stamens (anterior on the left, posterior on the right) and staminode; **M**. Anthers; **N**. Ovary and style; **O**. Stigma; **P**. Disk and ovary; **Q**. Longitudinally dissected ovary. Photos F, K, O, Q by D.V. Hai and A–E, G–J, L–N, P by B.H. Quang based on *B.H. Quang et al. VAST-PQ 05*.

#### Description.

***Herbs*** to 1.2 m tall, usually branching. ***Stems*** stout, terete, pubescent, green. ***Leaves*** opposite; petiole 3.5–7.5 cm long, pubescent, green; blade elliptic, 12–18 × 5–8 cm, base cuneate, margin entire or slightly sinuate, apex acute; blade adaxially dark green, pubescent, abaxially pale green, densely pubescent, especially on midvein and secondary veins; secondary veins 6–9 on each side of the midvein, netted near the margin. ***Racemes*** terminal and axillary, dense, 7–9 cm long, many-flowered; peduncle and rachis pubescent, green; bracts lanceolate to ovate, 8–20 × 3–10 mm, green, margin ciliate, apex acute, adaxially puberulent, abaxially pubescent, with 2–3 pairs of secondary veins; bracteoles (2 per flower) opposite, attached to the pedicel just below the calyx, narrowly oblanceolate to linear, 12–13 × 2–3 mm, margin ciliate, apex acute, adaxially puberulent, abaxially pubescent. ***Pedicel*** 2–3 mm long, pubescent, green. ***Flower*** weakly zygomorphic. ***Calyx*** 5-lobed nearly to the base; lobes linear, adaxially puberulent, abaxially pubescent, margin ciliate, unequal in size; unpaired (adaxial) lobe and lobes of the abaxial pair 15.0–15.5 × 2 mm, lobes of the adaxial pair 14.0–14.5 × 1.0–1.5 mm. ***Corolla*** ca. 3 cm long, outside shortly pubescent, inside shortly pubescent and hirsute below stamen bases, outside bright purple except for whitish proximal portion of the tube, inside bright purple except for pale purple adaxial side (2 lobes and sector of the tube), outside and inside with ca. 20 dark longitudinal lines on the tube; tube with a cylindrical proximal portion 9 mm long and 2.0–2.5 mm in diameter, widening to a cylindrical distal portion 15–16 mm long and 5.0–5.5 mm in diameter, slightly bent to the adaxial side at the transitional level; lobes 5, slightly unequal, oblong to suborbicular, ca. 5 mm in diameter. ***Stamens*** 4, didynamous, inserted at the transition between the narrow and wide portion of the corolla tube, included, white; posterior (adaxial) stamens 11–12 mm long, anterior (abaxial) stamens 14–15 mm long; filaments almost straight but bent downwards near their apices, glandular hairy (denser so towards apex); anthers with broad connectives and deeply separated thecae; thecae ca. 2 × 1 mm, margin glandular hairy; thecae connected in pairs; staminode 3.5–4.0 mm long, glabrous, light brown. ***Disk*** annular, pale green, smooth and lustrous. ***Ovary*** 2.5–3.0 mm long, ovoid-oblong, glabrous, green, bilocular; style appressed to the adaxial side of the corolla tube, near the base constricted by the bases of the stamen filaments, 2.5–2.8 cm long, glabrous, white; stigma unequally 2-lobed, slightly exserted; adaxial stigma lobe 2.5 mm long, apex serrate; abaxial stigma lobe 3 mm long, apex serrate. ***Capsule*** oblong, 8–9 × 3 mm, glabrous, 56–60-seeded; seeds oblong to narrowly ovoid, compressed, 0.9–1.0 × 0.5 mm, alveolate, pubescent.

**Figure 5.**
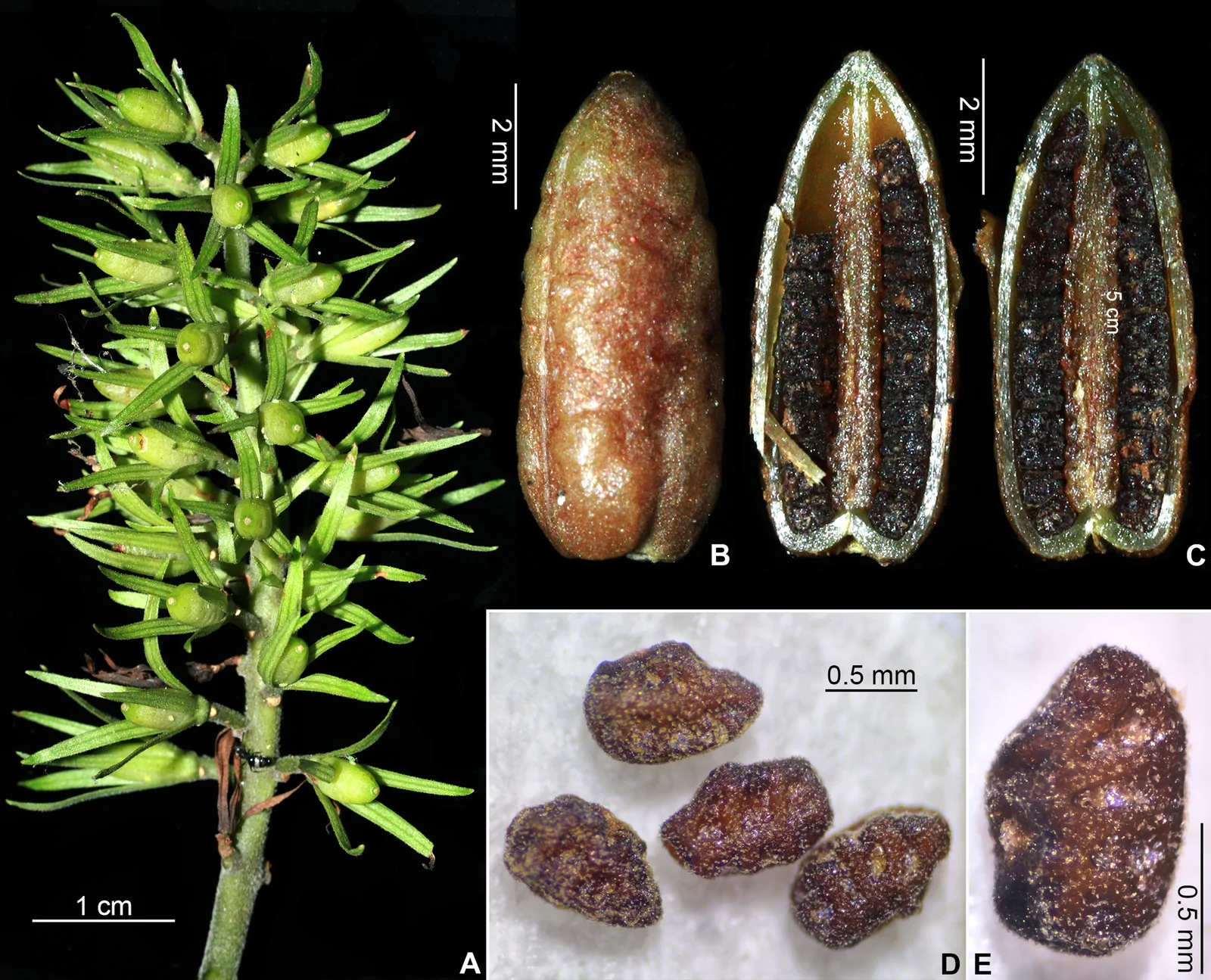
*Staurogyne purpurea*, fruit morphology. **A**. Infructescence; **B**. Fruit; **C**. Artificially opened fruit; **D, E**. Seeds. Photos by D.V. Hai based on *Hai, Sinh DVH 469*.

#### Distribution and habitat.

*Staurogyne purpurea* is currently known from Bat Dai Son Nature Reserve and Phong Quang Nature Reserve in Tuy­en Quang [previously Ha Giang] Province, Vietnam. It grows under the shade of very humid primary evergreen broad-leaved forests, and on steep rocky slopes of stream valleys composed of stratified, highly eroded limestone, at elevations of 800–1200 m.

**Figure 6.**
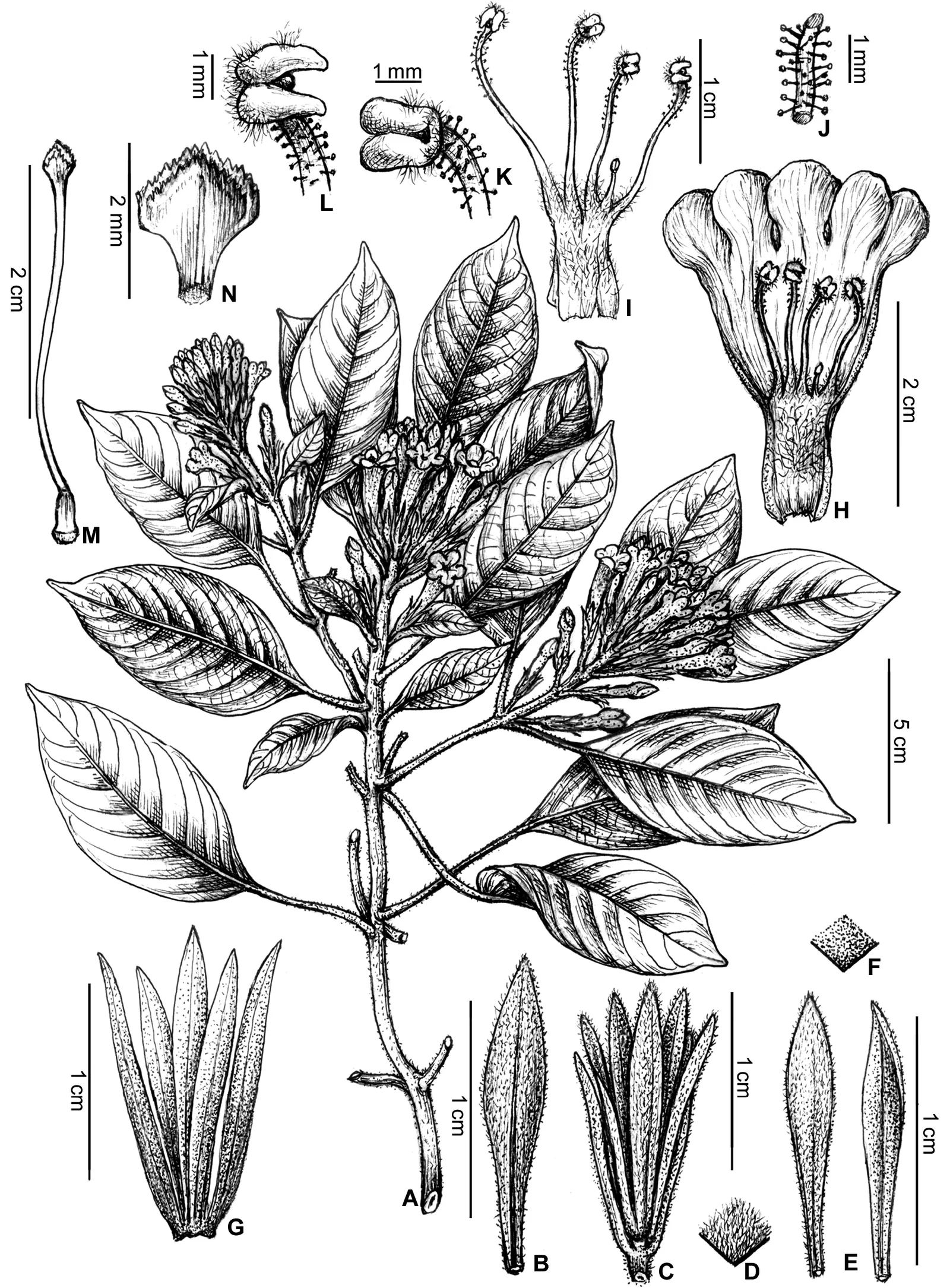
*Staurogyne purpurea*. **A**. Flowering Branch; **B**. Bract (abaxial view); **C**. Bracteoles and calyx; **D**. Outer surface of the bracts and calyx, showing hairs; **E**. Bracteoles; **F**. Inner surface hairs of the bracts and calyx; **G**. Artificially opened calyx; **H**. Artificially opened corolla; **I**. Stamens (anterior on the left, posterior on the right) and staminode; **J**. Portion of stamen filament, showing indumentum; **K**. Anther (abaxial view); **L**. Anther (adaxial view); **M**. Ovary and style; **N**. Stigma. Drawn by Le Kim Chi based on *B.H. Quang et al. VAST-PQ 05*.

#### Phenology.

Flowering in April; fruiting in June–July.

#### Etymology.

The epithet “*purpurea*” apparently refers to the purple corolla of this species.

#### Vernacular name.

Vietnamese (proposed here): *Nhụy thập hồng*.

#### Conservation status.

*Staurogyne purpurea* was collected from two localities in Tuyen Quang [previously Ha Giang] Province, northern Vietnam. Both localities are within legally protected areas, namely the Bat Dai Son Nature Reserve and Phong Quang Nature Reserve. The new species grows at undisturbed sites, and no anthropogenic threat has been detected. Pop­ulation assessment in the field has never been performed. According to our prediction, there are limestone mountains with habitat conditions similar to those of the type locality in Tuyen Quang, Cao Bang, and Lang Son provinces of northern Vietnam, in which more populations of the species probably occur. Therefore, we preliminarily assess its status as ‘Data Deficient’ (DD) according to the IUCN Red List Categories and Criteria (IUCN 2024).

#### Additional specimens examined.

Vietnam • Tuyen Quang Province [previously Ha Giang Province, Quan Ba District]: Tung Vai Commune, Thang village, Bat Dai Son Nature Reserve, around point 21°03'13.4"N, 104°51'48.8"E, at elevation 1050–1150 m a.s.l., steep slopes of streams valleys composed of stratified highly eroded limestone, primary evergreen broad-leaved very humid forests, terrestrial herb to 1 m tall on shady rocky slopes, flower purple, 21 April 2018, *L. Averyanov, K.S. Nguyen, N.T. Hiep, N.Q. Hieu, C.Q. Ngan, T. Maisak, VR 503* (paratypes: HN, LE LE01049245, LE01049485); • ibid., 4 April 2024, *B.H. Quang, T.D. Binh BHQ 1056* (HN); • Tuyen Quang Province [previously Ha Giang Province, Vi Xuyen District]: Minh Tan Commune, Phong Quang Nature Reserve, 22°55'16.5"N, 104°53'57.0"E, elev. 773 m, 13 April 2025, *B.H. Quang, N.T.T. Huong, D.T. Hoan, L.N. Han, N.T. Thuy, T.D. Binh VAST-PQ 05* (HN); • ibid., 28 June 2026, *D.V. Hai, V.S. Sinh DVH 469* (HN).

#### Taxonomic notes.

Since Averyanov et al. (2020; figs 718–720) provided only a brief description of this species, the present paper offers its first comprehensive account, including detailed morphological data and analytical illustrations. Morphologically, *S. purpurea* is most similar to *S. caobangensis* D.V.Hai & Joongku Lee, endemic to northern Vietnam, and *S. vicina* Benoist, known from Yunnan (China) and northern Vietnam, but differs from both species by its leaf blades 12–18 cm long (vs. 9–10 cm long in *S. caobangensis* and 6–15 cm long in *S. vicina*), lanceolate to ovate bracts (vs. ovate to deltoid and linear, respectively), and narrowly oblanceolate to linear bracteoles (vs. ovate to obovate and lanceolate or linear, respectively). It is further distinguished by its bright purple corolla (vs. pure white in both allied species). A detailed comparison of these three species is provided in Table 2.

**Table 2.** Morphological comparison of *Staurogyne purpurea*, *S. caobangensis*, and *S. vicina*.

**Characters**	***S. purpurea***	***S. caobangensis***	***S. vicina***
References	Averyanov et al. (2020), this study	Hai et al. (2018)	Benoist (1933, 1935), Chen et al. (2006), Hu et al. (2011) and our study of the type specimen P00650267 (http://coldb.mnhn.fr/catalognumber/mnhn/p/p00650267)
Plant height	up to 120 cm	up to 40 cm	up to 50 cm
Stem branching	usually branched	usually unbranched	usually unbranched
Petiole length	3.5–7.5 cm	2.5–3.5 cm	2–4 cm
Leaf blade: size	12–18 × 5–8 cm	9–10 × 5–6 cm	6–15 × 3–7 cm
Leaf blade: indumentum	adaxially pubescent, abaxially densely pubescent, especially along the midvein and secondary veins	adaxially pubescent, abaxially pubescent and sparsely hirsute along veins	both surfaces glabrous except for sparse pubescence along veins
Leaf blade: number of pairs of secondary veins	6–9	9–10	10–12
Inflorescence: position	terminal or axillary	terminal or axillary	terminal
Inflorescence: density	dense	dense	lax
Peduncle and rachis: indumentum	pubescent	pubescent	glabrous
Bracts: shape	lanceolate to ovate	ovate to deltoid	linear
Bracts: size	8–20 × 3–10 mm	14–15 × 11–12 mm	4–7 × ca. 1 mm
Bracts: indumentum	abaxially pubescent, adaxially puberulent, margin ciliate	pubescent on both sides, margin ciliate	glabrous or subglabrous
Bracteoles: shape	narrowly oblanceolate to linear	oval to obovate	lanceolate or linear
Bracteoles: size	12–13 × 2–3 mm	13–14 × 8–9 mm	2–3 × ca. 1 mm
Bracteoles: indumentum	adaxially puberulent, abaxially pubescent, margin ciliate	pubescent on both sides, margin ciliate	glabrous or subglabrous
Pedicel: length	2–3 mm	1–1.5 mm	2–3 mm
Pedicel: indumentum	pubescent	tomentose	glabrous or pubescent
Calyx: length	14–16 mm	13–14 mm	5–7 mm
Calyx: indumentum	adaxially puberulent, abaxially pubescent, margin ciliate	pubescent on both sides, margin ciliate	glabrous
Corolla: color	bright purple	white	white
Corolla: length	ca. 3 cm	ca. 3 cm	1.8–2.2 cm
Corolla: size of lobes	ca. 4 mm in diameter	ca. 6 mm in diameter	ca. 4 × 3 mm
Stamens: indumentum	glabrous	glandular hairy	glabrous
Anther thecae: length	2.0 mm	1.5 mm	ca. 1 mm
Anther thecae: indumentum	margin glandular hairy	margin hispid	glabrous
Staminode length	3.0–3.5 mm	ca. 0.5 mm	unknown
Style: length	2.5–2.8 cm	2.2–2.5 cm	1.0–1.2 cm
Style: indumentum	glabrous	glandular hairy at the base and glabrous otherwise	glabrous
Stigma: shape	2-lobed	2-lobed	2-cleft
Stigma: size	upper lobe 3 mm long, lower lobe 2.5 mm long	upper lobe 3 mm long, lower lobe 2 mm long	1 mm long
Stigma: shape of apices	serrate in both lobes	serrate in upper lobe, verrucose in lower lobe	verrucose in both lobes
Fruit: indumentum	glabrous	puberulent	glabrous

## Supplementary Material

XML Treatment for Staurogyne konchurangensis

XML Treatment for Staurogyne purpurea

## Additional information

### Artificial Intelligence (AI) use

The authors accept full responsibility for the content of the manuscript, including the disclosure of any use of AI.

No AI tools were used in the preparation of this manuscript.

### Author ORCIDs

L.N. Han https://orcid.org/0000-0003-2657-5091

B.H. Quang https://orcid.org/0000-0001-6878-7514

K.S. Nguyen https://orcid.org/0000-0001-5171-4140

M.S. Nuraliev https://orcid.org/0000-0001-8291-2633

R.K. Choudhary https://orcid.org/0000-0001-6250-4624

D.T. Hoan https://orcid.org/0000-0002-4298-5720

D.V. Hai https://orcid.org/0000-0001-7841-0585

### Data availability

All of the data that support the findings of this study are available in the main text.
